# Capture and express, question and understand: Gloves in gestural electronic music performance

**DOI:** 10.1017/wtc.2022.3

**Published:** 2022-05-05

**Authors:** Jan Schacher

**Affiliations:** 1 Institute for Computer Music and Sound Technology, Zurich University of the Arts, Zurich, Switzerland; 2 Centre for Music and Technology, Sibelius Academy, University of the Arts Helsinki, Helsinki, Finland

**Keywords:** embodiment, expressive interaction, gestural electronic music, gloves, methodology reflection, research approaches

## Abstract

Gesture-based musical performance with on-body sensing represents a particular case of wearable connection. Gloves and hand-sensing interfaces connected to real-time digital sound production and transformation processes enable empty-handed, expressive musical performance styles. In this article, the origins and developments of this practice as well as a specific use case are investigated. By taking the technical, cognitive, and cultural dimensions of this media performance as foundation, a reflection on the value, limitations, and opportunities of computational approaches to movement translation and analysis is carried out. The insights uncover how the multilayered, complex artistic situations produced by these performances are rich in intersections and represent potent amplifiers for investigating corporeal presence and affective engagement. This allows to identify problems and opportunities of existing research approaches and core issues to be solved in the domain of movement, music, interaction technology, and performance research.

## Introduction

Effort is consciousness of our struggles with the matter of music, music without matter can not sound (Ryan, 1992).

Traditionally, music performance comprises a physical action that injects energy into a resonating instruments. Electronic sound production and digital music processes have disassociated this link and imposed the necessity to redefine and formalize the connections between musician’s intentions and sound producing devices. Historically, the creators of electronic instruments had to deal with this issue, which led to the development of keyboards, controllers, algorithms, and automations of all sorts. Once accepted models were found, alternative modes could begin to take advantage of the separation between physical action and sound process. Gestural electronic music performance arose as one possible approach to tackle this challenge and represents the condensation of the development in terms of interactivity and real-time performability of digital processes. The performers were trying to bring the body back into electronic music, which had become increasingly dissociated, especially in works with fixed media such as loudspeaker orchestras and tape pieces (Waters, 2000; Cypess and Kemper, 2018).

This article traces one specific lineage of interfaces for music making: It looks at glove instruments, their strengths and limitations, and investigates early designers’ aspirations and a few concrete implementations. By describing and analyzing the practice of these pioneers, as well as the author’s own approach, questions about effort, tangibility, gesturality, and the understanding of movement qualities can be addressed. By focusing on the Dutch experimental tradition, a continuous development and common paradigm can be made evident. The trajectory begins with Michel Waisvisz’s “The Hands,” from the mid-1980s, moves on to Laetita Sonami’s “Lady’s Glove” in the 1990s and the 2000s, and also covers Atau Tanaka’s Bio-Muse performances using electromyogram (EMG) signals of hand gestures. This background provides a frame within which to place the author’s own experience of two decades spent with the development, composition, and performance of gestural electronic music using sensor gloves. This practice serves as a use case to exemplify a gesture-based approach, and to provide knowledge from inside the development, composition, and performance processes. From this perspective, it should become possible to develop a structured approach to addressing fundamental questions related to research about gestural music performance, movement computation, and affective embodied relationships to performance in general.

### Underlying Issues (Overlaid)

When performing with gloves, a number of pressing issues come to light that expose the topics relevant to the wider domain of wearable interaction and computing. These issues refer to a wide array of domains, such as cognitive sciences, physiology and ergonomics research, history of science and technology studies, media studies, human–computer interaction (HCI), as well as embodiment and enaction, to name a few. Without the necessary space to dive into these complex matters in this article, their important contributions and essential connections to the topic at hand are acknowledged, and a very brief link is offered in this section; some of their deeper implications are discussed in the second half of the article.

In the case of performing music, which differs sharply from more common uses of wearable technologies, the questions are how the link to the intentionality of musician is established, how multiple layers of attention can be directed, and how physiological and body-schematic conditioning influences the usability as well as the legibility of the gesture interaction.

Carrying out empty-handed (Héon-Morissette, 2012) actions with overlaid sensing and mapping them to digital processes produces a split, multilayered attention in the performer. All our actions are constituted of embodied, preconscious behavior patterns, that is, well trained and ingrained body schemata (Gallagher, 2005). In the case of wearable performance, these patterns are recontextualized and tend to become explicit and conscious while they are adapted to accommodate the action space of the sensing device and the digital sound processes.

Similar to many complex corporeal tasks, by leveraging the body’s capabilities to reinscribe and overwrite established patterns with new schemata, interactions with on-body sensing devices need to be learned through repeated, integrating practicing. The goal of transparent, fluid interaction without any additional cognitive load can only be achieved with extensive training (cf. statements by Waisvisz and Sonami in the “The Dutch Touch and Beyond” section).

The development of methods for using on-body sensing in performance happens through layers of integration. A tool or instrument relationship needs to be established, and metaphors of interaction are designed. The high-dimensional control spaces of hand motions need to be reduced and remapped to a space of actions and processes that resemble the habitual relationship we have with objects in the natural world. Ultimately, the main reference system remains conventional tool use with technical devices such as turntables, typewriters, and laboratory equipment (Simondon, 1958; Eshun, 1998; Rheinberger, 2021).

The placement of sensors on the body for measuring movements and postures generates an augmented gesture space (Agamben, 2000; Godøy and Leman, 2010). As a consequence, semantic or functional enrichment occurs in an already densely packed bodily domain of action, interaction, expression, and signification. Wearable interfaces tie in with the body in comparatively narrow affordance spaces (Gibson, 1977)—contrary to conventional control interfaces, the wearable relationship superimposes technical structure onto the bodily space. One of the differences with conventional tools and a central difficulty is that—once put into place—the relation cannot be fundamentally changed, only modulated, engaged with, or dissociated from.

## Background/Context

Part of field of reference of this practice is research on the signification of *Gesture* from the linguistic perspective (Kendon, 2004; McNeill, 2005; Gritten and King, 2006), and in particular research related to music making (Jensenius et al., 2010; Gritten and King, 2011). Other directly related domains are: on-body sensing for performance in the disciplines of dance (Siegel and Jacobsen, 1998); for music with full-body engagement (Schacher and Stoecklin, 2010; Todoroff, 2011; Schacher et al., 2016); remote sensing of the entire body or just the hands for musical purposes (Bevilacqua et al., 2001; Hantrakul and Kaczmarek, 2014; Tits et al., 2016; Jakubowski et al., 2017); or hand sensing with on-body sensors (empty-handed or functional triggering actions; Brown et al., 2020).

In the music technology field, this background concerns instrument developments with new paradigms of interaction that are closely tied to the hands and manual dexterous actions; this is well represented in the New Interfaces for Musical Expression (NIME) community (Jensenius and Lyons, 2017). Overall, the practice inherits from all major technological developments of the past century, ranging from those involving electricity, to electronics, computers (Mathews, 1963), the now ubiquitous digital signal processing and modeling (Impett, 2001; Alaoui, 2013; van Eck, 2017), and finally machine learning (Fiebrink et al., 2011; Françoise et al., 2014).

### Historical Practices

The use of gestural interfaces for music performance is as old as electronic music production itself. The Theremin (Theremin and Petrishev, 1996), the Trautonium (Sala, 1950; Patteson, 2016), and the Ondes Martenot (Vaiedelich and Quartier, 2013) represent early twentieth-century approaches to synthetic sound production with gestural (instrumental) playing modes. In many cases, conventional musical instruments provided the template for modes of playing and interacting with these synthetic sound sources. Physical instruments represented the frame of reference for the action patterns and nuances of expression that the new instruments strived to recreate.

In the 1980s, the MIDI protocol emerged as the standard that allowed keyboards and synthesizers from different manufacturers to interconnect and operate in as unified whole. This standard is still in use, implemented in almost all musical devices and software, and continues to be developed today (Diduck, 2015). Around the same time in the 1980s, the video and arcade games began introducing interaction with game controllers that simulated gestures and actions with real-world objects, such as guns, golf clubs, and so on, going well beyond the computer interaction modes that had been pioneered in the late 1960s (Engelbart, 1968; Engelbart and English, 1968), designed for office use (Buxton and Myers, 1986). In parallel, with the first wave of VR, sensing of hand gestures, such as grasping, turning, and moving, became a desirable feature to increase engagement and the sense of immersion through believable interaction with and in the virtual world (Bridgewater and Griffin, 1993). Musically, early on, the idea of gloves for synthesis, specifically vocal synthesis in sign language application, was explored with “GloveTalk” (Fels and Hinton, 1993), and the Nintendo game peripheral “Powergloves” that became the prime example of a commodified device, later dismembered for its flex sensors (Mulder, 1994a).

The use of interfaces for sound control emerged with the advent of digital systems that allowed the separation of the sound-producing and sound-controlling parts of the instruments (Mathews, 1991; Rich, 1991). The first sensing gloves originated at that time, and since then, pursuing the goal of natural gestural interaction with digital worlds and artefacts produces new iterations of this paradigm in recurring intervals (Clark et al., 1989). Already at that time, the separation between body and interface was an important topic and explored in various types of digital performance configurations, for example, in the audiovisual performances of Ostertag (2002). Detailed reflections on the historical evolution and perspectives on computer music performance can also be found in Wessel and Wright (2002).

In parallel developments, composers and choreographers started to explore the emerging technology for their interpretation potential. Thierry deMey’s “Light Music” from 2004 is an exemplar for a composed piece mixing gestural music and image, and represents an iconic implementation of empty-handed gestures with “outside-in” camera tracking (Mulder, 1994b; Héon-Morissette, 2012, p. 11).^1^ In the 2010s, projects of note were the Synekine research that addressed synesthesia and proprioception as well as HCI topics (Beller, 2014), and the MiMu Gloves by pop-artist Imogen Heap (Mitchell et al., 2012), the only development of sensing gloves that reached the maturity of a stable commercial product.^2^ The African-American media artist and musician Onyx Ashanti developed an idiosyncratic gestural performance style with DIY interfaces that carries a strong afro-futurist influence (Zamalin, 2019).^3^ Finally, from a wider pop-cultural perspective, Spielberg’s science-fiction film “Minority Report” from 2002 established a strong cultural icon of gestural interaction with empty-handed spatial interaction (Russell and Yarosh, 2018).

In the next section, this article will address in more detail the “Dutch School,” which became since the 1980s an important reference for interfaces and real-time software developments for gesture-based performances in electronic music.

## The Dutch Touch and Beyond

But these “handles” are just as useful for the development or discovery of the piece as for the performance itself. In fact, the physicality of the performance interface gives definition to the modelling process itself (Ryan, 1992).

The main context and lineage discussed in this article originates from the *Studio Voor Electro-Instrumentale Muziek (STEIM)* in Amsterdam and the Dutch experimental music context in general.^4^ Three performers and their instruments shall serve as examples of developments around “the physicality of the performance interface,” its specificity, and the artistic approaches to solving the question of modeling the process. The instrument and software developments were of course not carried out by these musicians alone, and the reflection on electronic music instrument construction by Bongers (2007) provides a comprehensive witness statement about the STEIM developments from their “luthier’s” point of view (Jordà Puig, 2005).

### Michel Waisvisz

A ground-breaking performer of gestural electronic music was Michel Waisvisz, in particular because the integration of his instrument on a body-image level was so evident. He constantly applied his instrument to artistic performances, developing a repertoire and style over an extended period of time. The mixture between instrumental control and physical movement, combined with direct treatment of vocal sounds, generated an expressive performance style that appealed on the physical, the corporeal, as well as on the musical level. His instrumental developments and compositional practices were based on an in-depth experience in open-form instant composition and theatrical performance (Bellona, 2017). His exploratory instrument-building ethos was guided more by the notion of direct physical interaction potential than maintaining an absolute control over the music. Waisvisz’s physical performance attitude was described by observers as resembling that of a boxer or a pilot (both were activities that he practiced at various points, e.g., flying a motorized paraglider). He considered his musical roles to be simultaneously that of composer, interpreter, and conductor (Otto, 2016, p. 128).^5^

The instrument he was most known for was a gestural MIDI controller to be worn on both hands, aptly named “The Hands” (Waisvisz, 1985, p. 9, 2003). The combined controller and software instrument—one of the most deliberate digital musical instruments (DMIs)^6^ demanding the full integration of both parts—was developed in the early 1980s and experienced three revisions, yet stayed fundamentally the same throughout the two decades of its use (Krefeld and Waisvisz, 1990; Torre et al., 2016; Bellona, 2017). Waisvisz emphasized the need for this consistency and argued that he “wanted it to be able to become second nature” (in an interview; Otto, 2016, p. 134). Waisvisz’s influence as a performer and director of the STEIM foundation in Amsterdam during more than two formative decades influenced many artists, guiding them to pursue the discovery of self-built, idiosyncratic instruments.^7^

### Laetitia Sonami

French-American artist Laetitia Sonami was directly connected to the Dutch school at STEIM. With the technicians of the foundation, she codeveloped her glove interfaces, also in several iterations. Her main instrument, the “Lady’s Glove” extended and modified the very pragmatic and technical instrument used by Waisvisz and added a theatrical, cultural, and stylistic dimension. Her musical approach and compositional strategies remained in the exploratory attitude, where the exact form of a piece was newly created for each performance. During the long-term development of her practice, she also deployed her voice with texts, as well as various types of sound materials that she accessed, modulated, and composed with the gestures afforded by her system. She navigated the prepared compositional structures “as a set of templates or architectural playing fields” and negotiated them in a kind of struggle, which she describes as “bull riding … when performing with this system” (Sonami, 2006). Using the constraints of the system became part of the strategy that she expressed in a potent way: “I am tethered to sounds caged in laptops … trying to make the dance look effortless” (Sonami, 2007). This showed the need to obtain a handle on a complex, multidimensional, as well as technical action space. “The intention in building such a glove was to allow movement without spatial reference, and most importantly to allow for multiple, simultaneous controls” (Sonami, 2021). In her combination of glove interface and software instrument, she managed to configure a system that offered a field of possibilities, algorithms, sounds, and processes, ready to be shaped during the performance. She reached a level of body-instrument integration that was similar to that of Waisvisz; her glove instrument evidently produced a gesture space that afforded her a fluid bimanual, embodied expression. The combination of glove gestures with transformations of vocal sounds led to a type of performance that needed to be experienced directly, emphasizing the corporeal forms as expressive communications. For her, “the sounds are now ‘embodied,’ the controls intuitive, and the performance fluid. It has become a fine instrument” (Sonami, 2021).^8^

### Atau Tanaka

Active in the Dutch context in the 1990s and the early 2000s, the “Sensorband” was a group of musicians that explored interactions using various strategies for interfacing with sound processes (Bongers, 1998). The “Soundnet” (Jordà, 2005) was an embodied, room-size climbing instrument, exemplifying the use of sensing and interacting as a fundamental performance mode. The group was formed by Dutch musician Edwin van der Heide, Polish noise musician Zbigniew Karkowski, and Japanese sound designer and musician Tanaka (2000). The main gestural interface that Tanaka used in the group’s performances was the BioMuse system (Tanaka, 1993), an early EMG system (Tanaka and Knapp, 2002) that extracted MIDI information from the electrical signals produced by the forearm muscles. In a similar manner as the two previous artists, he developed and refined the performance system to comprise the entire chain from physical action to sound output: “The instrument … is made up of the specific placement of the electrode armbands, the BioMuse hardware, the firmware program running inside the BioMuse, the MAX software on the host computer post-processing the biosignal data, and the sound synthesis implemented in software in MSP and realized in hardware on the Macintosh. This instrument has remained more or less a constant” (Tanaka, 2000, p. 392). He presents a clear distinction between instrument and composition and like Waisvisz and Sonami emphasizes the need to keep the instrument constant. In his concept, the sensing hardware and the sensor conditioning, as well as the sound reproduction, belong to the instrument, while the specific mappings and sound synthesis processes are considered part of the composition: “The composition then is the choice and combination of different biosignal post-processing techniques as connected to various synthesis articulations, place[d] in context of a musical structure over time.” He acknowledges that his use of the BioMuse as gestural interface is quite physical and depends on empty-handed engagement: “The BioMuse is an abstraction of instrumental gesture in the absence of a physical object to articulate music through corporeal gesture” (Tanaka, 2000, p. 393). With the development of more accessible and wireless EMG systems, such as the Myo armbands (Nymoen et al., 2015; Rawat et al., 2016; Di Donato et al., 2018),^9^ Tanaka continues to compose pieces using this sensing modality^10^ and still performs himself.^11^ His attitude and musical interest is best summarized in the question he poses: “How can we best make use of multiple modes of interaction with gestural input to create rich musical results?” (Tanaka and Knapp, 2002).

## Performing “New Islands”

**Figure 1.**
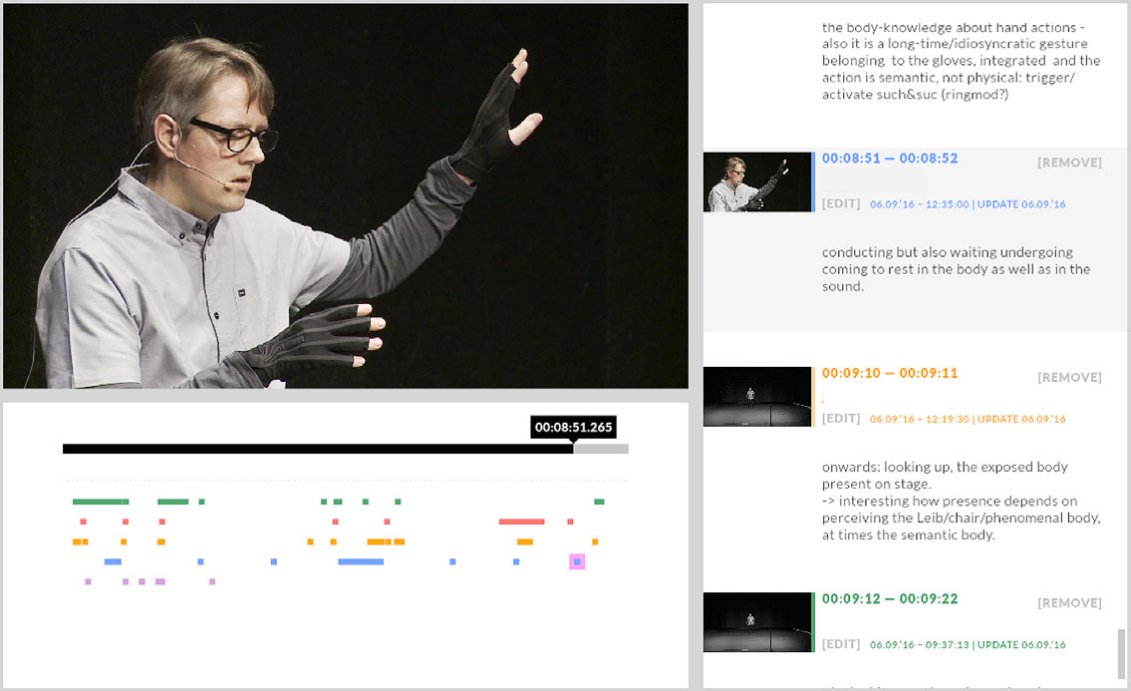
A performance of “new islands” by the author in the process of annotation. Detailed description and auto-ethnographical trace collections allow for in-depth understanding of effectiveness of composition and development strategies, bodily states, and proprioception as well as identification of decision moments in the flow of the performance (Software: Piecemaker to go “pm2go” by Motionbank [Forsythe, 2013] from 2016, since superseded by Motionbank’s online “Motion Bank System”).

The author’s own trajectory using sensor gloves begins in 2000 with the assembly of a first prototype. The wearable interface was originally intended for a specific application in the augmentation of conventional string instrument with hand-position sensing. This constraint informed the design of the glove with open fingertips, a feature found again in the commercial Mi.Mu gloves developed a decade later (see the “Technical Implementation” section for more details). The capabilities of combined finger position and attitude sensing (using flex sensors and an accelerometer, respectively) subsequently enabled the author to use the gloves in a number of different performance configurations beyond the initial live-electronics composition (Figure 1). A recurring configuration used the interface for gestural control of real-time motion graphics in live audiovisual performances (Schacher, 2007a, 2008).^12^ With the addition of a second identical glove, the use of gestures for audio spatialization became a research topic in its own right (Schacher, 2007b). A cycle of performance pieces and media composition was initiated in 2010 under the title “new islands” and serves to explore the possibilities of empty-handed, gestural electronic music performance (see below in the “Empty Handed Play” section).

### Technical Implementation

**Figure 2.**
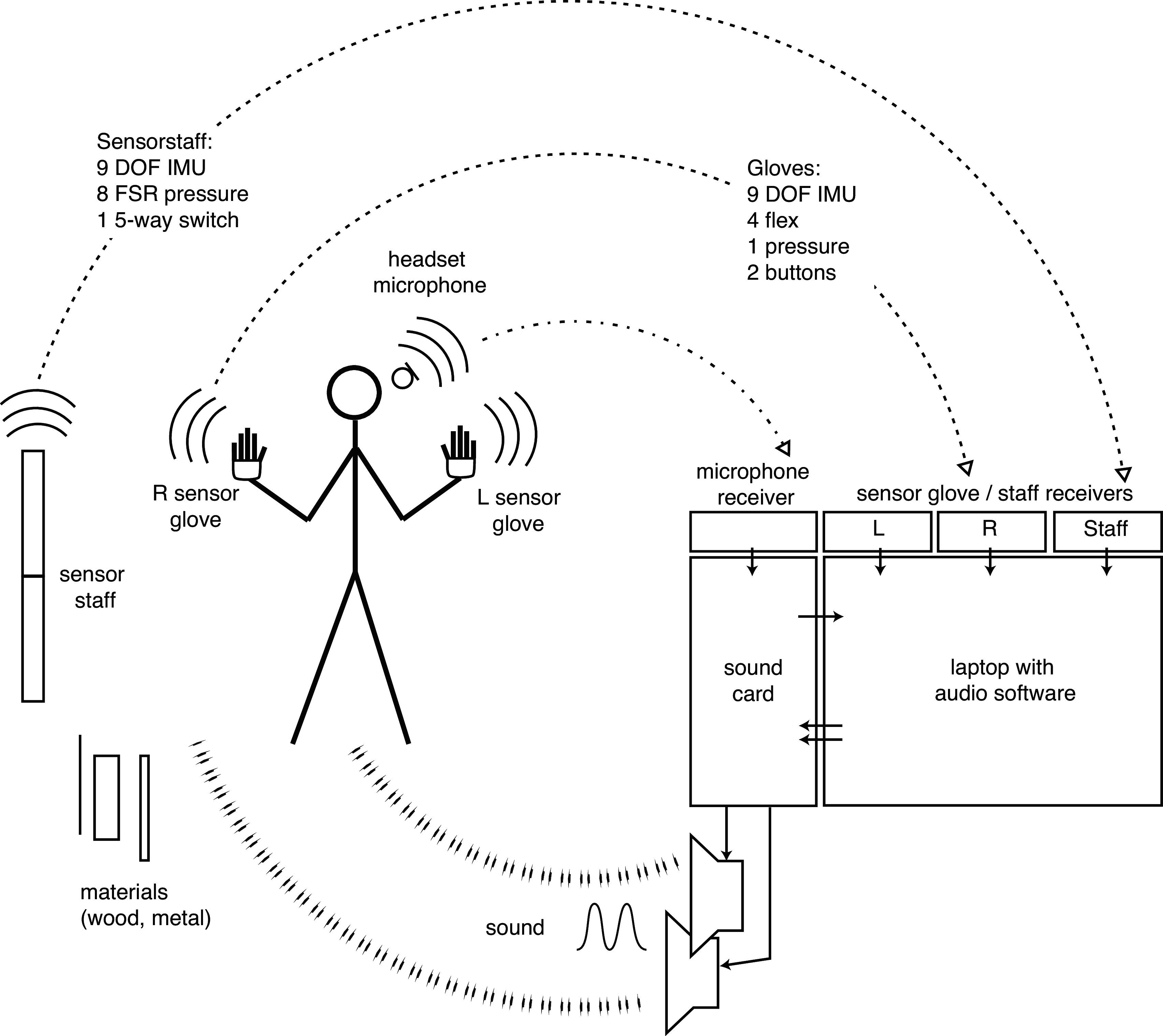
System overview for “new islands”: The performer is equipped with a wireless headset microphone and a pair of wireless sensor gloves; in addition, a wireless sensor staff and other materials such as woodblocks and metals are used. The other half of the system consists of the receivers, an audio card, and software systems for sound transformation processes. The sound outcomes influence the performer’s perception and decisions, thus closing the action-perception loop.

The basic idea of the empty-handed performance without an interposed screen is based on technical methods for exerting control or influencing sound processes without having recourse to the conventional cockpit-like control arrays or the traditional physical instrument (Schacher, 2019). The sensor-bearing gloves and at a later point a sensor-equipped staff-like interface (Schacher, 2013) enable the purely gesture-based modulation, navigation, and expression of musical materials without the need to directly access software and control parameters (see Figure 2).

The two sensor gloves were handmade between 2001 and 2005 and have stayed in a stable form since then. The conversion electronics and motion sensors are updated as the available microcontroller and wireless technology evolves: All the elements have become wireless, providing complete freedom of movement on stage.

The gloves are silk undergloves whose fingertips have been removed to enable tactile actions, such as playing a traditional instrument. Each glove carries a flex sensor on the four main fingers, which measures the overall bend of the finger. Placed on the back of each hand lies a nine-degree-of-freedom movement sensor. This sensor corresponds to those used in current smartphones and produces continuous three-dimensional data at a high sampling rate for acceleration, rotation, and the magnetic field as an orientation vector.

The accelerometer measures the energy of movements, and for static poses reports the pull of gravity and thus spatial orientation similar to a joystick. The gyroscope measures rotational speed and is particularly sensitive to flicking and twisting movements. The magnetic sensor, in combination with the gravity vector, is used as a digital compass, measuring the absolute orientation in relation to the external frame of reference.

A push button is located under the first finger of each hand, and on the thumb-facing side of each first finger lies a small pressure sensor. Finally, the cables leading to all of these sensors are collected on the back of the hand and connect to a bracelet that carries all the necessary conversion and communication electronics such as microcontroller, wireless module, and the rechargeable battery.

As seen in the instruments used by the three artists introduced in the background section, the complete gestural performance system is comprised of several components, both in hardware and software. In this case, the glove interfaces with wireless transmitters form the hardware part, whereas the sensor conditioning, mapping, and sound-producing algorithms in the software part complement the gloves to combine into a complete DMI. Stability and learnability of such a system represents a key characteristic necessary for the development of a long-term artistic trajectory.

The basic technical setting for the sound processing of the “new islands” cycle has not changed since the first performances in 2010. The microphone signal is routed through a sound card, gets transformed and augmented in software, and the resulting sounds are played back on a standard stereo PA-system, to be heard by both the performer and the audience (see Figure 2). The sound-processing software consists of a global audio-routing system feeding a number of modules, as well as a control layer that manages the overall settings. Most of the sensor inputs and mappings are executed separately in dedicated tools, the connection and translation of movements and action patterns, however, is integrated in one of the top layers of the software. The mappings and pattern recognition are of considerable importance for this system. Action patterns, for example, are used to trigger specific sound changes. One gesture that is easily recognized, for example, even in the video recordings, is the closing of the fist in a grasping movement and the corresponding release. Depending on the orientation and elevation of the hands, these actions have different functions and trigger discrete events: In one case, they activate the capturing of sound into one of the granular engines, and in another instance, they control the freezing of a reverb tail that provides textured sound surfaces. Other parameters are continuously affected by sensor values and are connected, for example, to the horizontal compass direction toward which a hand points.

**Figure 3.**
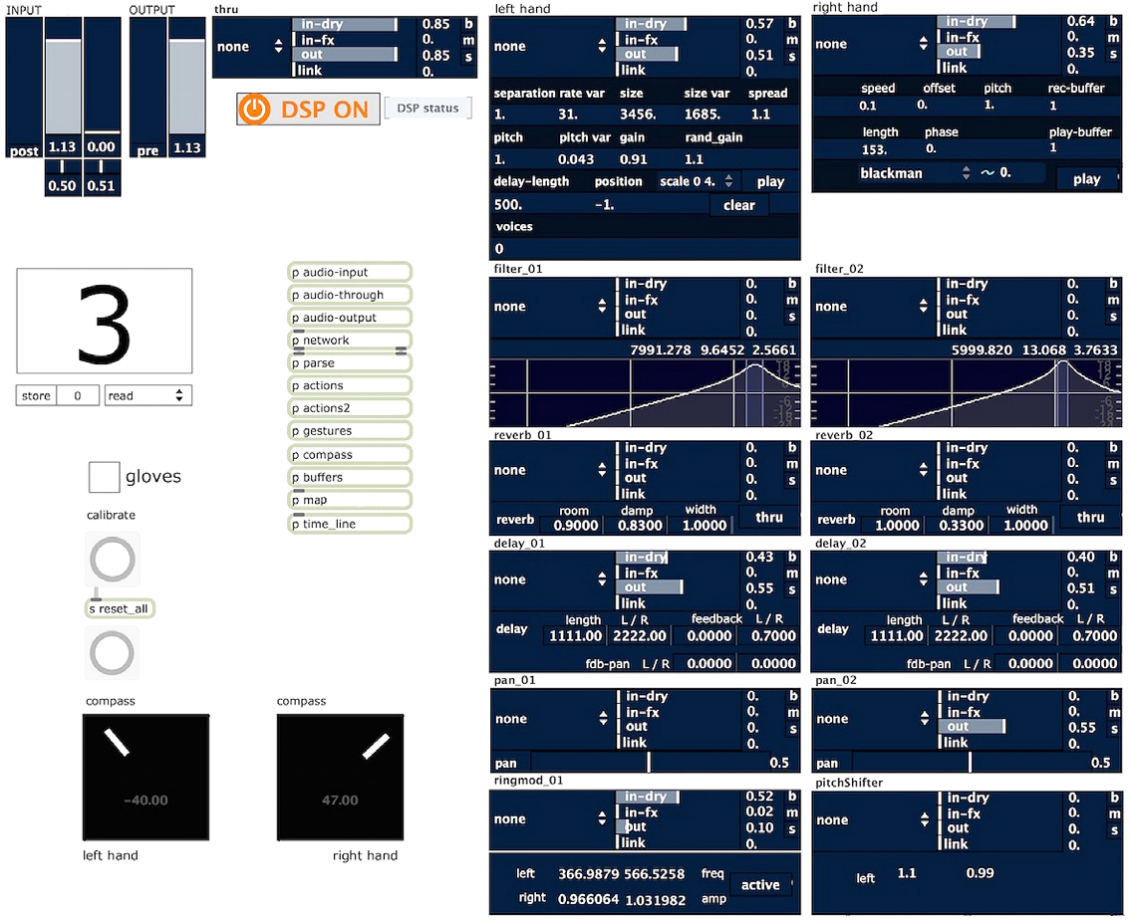
Screenshot of the software system as used in the performance of “new islands” in January 2016. Note the two compass displays with calibration on the lower left, the two vertical audio signal chains on the right (dark blue boxes, each parameter mappable), and the presence of network, parsing, actions, gestures, compass, mapping, and timeline data-treatment modules at the center (collapsed, not showing details). Dynamic audio signal routing and sensor parameter mapping is controlled by a preset system, displayed as large number, mapped and controllable by the gloves or a time system.

Part of the sound processing is split into two halves, corresponding to the two hands. A number of processing modules are identical for both hands, such as filters, reverbs, delays, and spatialization (see the two columns on the right in Figure 3). In addition, each hand is assigned to a specific process: The left hand starts, stops, and modulates a process that generates a rough, irregular, cut-up granulation; the right hand starts, stops, and modulates a long-duration process that mainly produces very fine-grained time-stretched playback of incoming sound fragments; in addition, the left hand controls a ring modulator, whereas the right hand affects a pitch shifting process.

### Empty Handed Play

From 2010 onward, the author’s creative focus shifted away from digital arts practices and visual stage and installation work, investigating gesture in a more systematic fashion. This research approach, combining instrumental interaction concepts with artistic research methods, led to the formulation of a compositional platform, which evolved into a series of pieces, or rather a recurring piece with an evolving format.

The piece *“new islands”* was premiered in 2011 and has been in ongoing development since, with one or two performances per year.^13^ The guiding principle for this composition is the exploration of sensor-based, gestural actions with empty-handed gestures, controlled with the aid of sensor gloves and cameras and a symbolic staff-shaped sensor instrument that restitutes the object character of an instrument without providing actual sound generation (Schacher, 2013). The current version of the piece concentrates on performing with a pair of sensor gloves and a wireless headset microphone. The piece can be regarded as a composition insofar as the real-time sound processing is highly structured and stable, but it could arguably also be regarded as a hybrid instrument encompassing the gloves, their mapping, and the digital sound processing. The structure of the actions, the intended performance energy, and the resulting forms are (re-)created every time, and have evolved on some levels, while in order respects they have remained stable.

The evolution of the piece and the way it “feels” to the author during performance provides a relevant connection to both the “enactive” and the neuroscientific perspectives (Schacher and Neff, 2016b). The insights and reflections gained about the corporeal states inform on a fundamental level how the piece gets structured. These difference become visible when watching a documentation video of several of the performances spanning 4 years. Mixed-method qualitative approaches, such as autoethnographical trace-collection (Ellis and Bochner, 2000; Denzin, 2014) and grounded-theory terminology developments (Glaser and Strauss, 1967; Charmaz, 2006), have formed part of the analytical processes carried out by the author in order to more closely understand the states, impulses, and trajectories that the performances have taken and how they have evolved over time.

The reduction of instrumental elements and the developing clarity of the gestural actions across this process on the one hand, and the increasing ease and expertise of execution in each performance on the other, are direct consequences of a stable musical framework, within which experiences have accumulated. The reflections about corporeal presence and its affective power also led the author to concentrating on a movement repertoire and sound language that emphasizes the immediacy of decision-making during performance. In order to leverage the skills and expertise of the performer and at the same time leave enough space for an exploratory attitude, key aspects or principles of the “composition,” such as the stability of mapping (Schacher et al., 2014), keeping manageable the size of the mental map of sound processes, and an independence from visual representation, have to be respected. This simultaneous fixing and opening of the compositional framework reflects the acquisition of skills and expertise on a corporeal, an experiential, a purely technical, and even an instrumental level, as well as the sharpening of the aesthetic, performative, and musical intent.

Through these artistic development processes, four temporal frames of expertise and experiential stabilization can be observed (Schacher and Neff, 2016a). The experience over time by the musician of how the mental map of the piece evolves and how the attitude in the playing of this piece changes indicates that a settling or crystallization process has occurred. This long-term solidification happens to a lesser degree for the audience as well: Throughout the performance, the principles, actions, and the ways the sounds and actions correlate are first perceived, then learned, and then fully recognized. The analogous process within the duration of a single performance is based on cultural and social assets (Rancière, 2009), but also on fundamental corporeal intersubjective identifications (Proust, 2003), and is necessary to produce affective impact (Russell, 1980). For the performer, thanks to the stable elements in the composition, his or her body remembers how it *feels* to gesture with these sound processes. For the performer, the sensor mapping and the mental map that are necessary to navigate the “instrument” or “piece” appear to have become imprinted—at least to some extent—and the corporeal impulses for certain gestures or movements during the performance start to resemble those that occur on a traditional instrument. For the listener-viewer who has witnessed *several* of these performances, the noticeable difference in the quality of performance—even if not specifically identifiable—indicates a similar settling; the situation becomes familiar, and the expected actions and sounds fall within a known field of possibilities. This familiarity comes from the stable and recurring parts of the “composition” and the unchanged stage situation that retains the same frontal setting for each performance. The familiarity is only to be relativized when the character of the piece changes from one performance to another, due to evolving artistic concepts used for the “narrative” aspects of the work. Thus, the effect of this stabilization also influences the perceptual integration of elements for the audience.

## Wearable Connections

As we have seen and has been articulated by the artists introduced above, on-body sensing and wearable controllers establish a particular link between the performing subject and their “instrument.” Through the sensors, gestures get captured, and at the same time, the performer gets tethered and tied in a close relation to the sound producing apparatus (Agamben, 2009). In this aggregate state, the artist’s intentionality gets filtered through a narrow channel of technical sensing modalities, discrete input devices, and observation and analysis models which attempt to regenerate a differentiated gesture vocabulary from limited data streams.

In a mutual embrace, the skin-tight connection engenders embodied transfer capabilities, which have the potential to translate from natural actions to augmented and overlaid interactions. Compared to conventional instruments and the evident object relationship they enable with the performers (Foucault, 1982), the skin-tight, closely molded, and nonremovable overlay of gloves and empty-handed interfaces generates a transparent movement and action twin that fuses with the body schema rather than remaining an external object (Gallagher, 2003). The artist finds herself in a situation of multilayered, split attention between body and apparatus, or—in the best of cases—in a state of pure, nontechnical movement expression, capable of intuitive (re)actions not imposed by technical concerns.

Based on the historical background and the specific practices shown thus far, the following sections will attempt to address conceptual questions that arise from putting sensors on bodies. The issues extend beyond the domains of musicianship and instrument building to address questions about the relationship between body and presence, movements and their qualities, and gloves as wearable technology.

### Shared Spaces

Musical interaction with empty-handed glove connection represents a specific paradigm of instrumental play. In addition to the on-body sensing part, the instrument usually also features a digital sound-producing part that is coupled through an interpretation layer. In this configuration, the artist remains shielded from the purely technical control surface and deals with an interface directly situated on their body. This surface merges or at least blends the technical and corporeal planes, overlaying sensors and the skin of the performer, and through this closeness has the potential to become perceptually transparent, not interfering any longer in the space between the performer and the audience. This quality enables physical presence in front of the audience to become one of the central aspects of the performance. No instrument or tool blocks or “protects” the musician from the audience; she faces her listeners in an unimpeded and direct relation. From a social standpoint, then, the empty-handed performance takes place in a shared space between performer and audience. This “corps-à-corps” (Nancy, 2008), body-to-body signaling and affecting (Gibbs, 2010) plays a central function in the effectiveness of these types of performances. The spectators experience not just musical content and a well-crafted time span with sounding content, they also enter into a mimetic and affective bodily exchange and (re)live on a prereflective level the impulses and efforts that the artists channel and shape into their musical expression. Through this connection, the artists become more than mere entertainers, and they establish an empathic, mimetic, and resonant space of shared experience (Hodkinson, 2020).

Interactive media artist and dancer Rokeby reflected on this already in the 1990s when he stated that “the artists’ role is to explore, but at the same time, question, challenge, and transform the technologies that they utilize” (Rokeby, 1995). When taken not just in the general sense of crafting interactive electronic music, but also with regard to the stage situation and the expression generated by empty-handed play, this statement obtains a particular significance. The complexity of dimensions at play, the lack of complete control, and at the same time the simplicity that empty-handed gestures enable (Ekman, 2015) have the potential to produce an intensity and richness in the performance that goes beyond the merely technical achievement and encompasses affective, emotional, and poetic domains.

What are the cultural norms evoked by these actions (referring to Waisvisz as a boxer or pilot in the “Michel Waisvisz” section)? How unambiguously do these gestures and corporeal expressions come across (referring to Tanaka moving his hands with effort in order to tense his forearm muscles in the “Atau Tanaka” section)? Are those movement patterns that are guided by the affordances of an on-body sensing system not always already technical control gestures (referring to Sonami’s and Waisvisz’s movements imposed by the use of ultrasound receivers for interhand distance sensing in the “Laetitia Sonami” section)?

### Movement Basics

Considered from a narrowly focused perspective, nontechnical elements of the gestural performance play an equally important role. Movements, actions, and gestures executed during the performance of gestural music can have different functions, and they can be expressive, symbolic, or purely functional (Godøy and Leman, 2010). Hands in particular have a special status, since they represent one of our central interfaces to take action on the material world. Hand gestures or actions are therefore always read as expressing an intention and as being teleologically aimed at some “thing” or process.

From the on-body sensing perspective, this strong perceptual attribution provides a clear context within which to operate: By obtaining kinematic data from hand positions (grasping and pointing); by measuring postural data of the hands (palms up/down), or pointing forward (signaling gestures); and by extracting dynamic data combined with positional data—when measured directly on the hands, these actions can become placeholders for the entire kinematic chain of bodily action. These placeholders signal the performer’s focus and are read as directed and intentional.

The dynamic aspects of hand movements carry expressive qualities in addition to functional and ergonomic energy trajectories (Viviani and Flash, 1995; Ryo et al., 2019); the gesture dynamics also carry the signifying aspects of a gesture. Through these dynamic profiles, even a task-oriented action obtains a signifying aspect in empty-handed gestural performance.

### Gloves and Wearables

Intersecting the spaces of hand gestures, the hand’s high number of degrees of freedom with the technical capabilities of sensing and mapping describes another peculiarity of gloves for performance. Compared to numerous technical interfaces, gloves provide the following characteristics:

- capturing bimanual dexterity;
- enabling fingertip control actions;
- measure of hand posture and hand position in space;
- alternative hand gestures, per hand or relative to each other;
- building on tool actions that are mostly manual (i.e., musical instruments as tool class);
- sensing modes are either egocentric inside-in or outside-in (on-body sensing vs. bimanual ultrasound or camera-based white glove capture); and
- leveraging imprinted patterns of hand-action expertise and dexterity both for the performers and the audience.

What is problematic in this categorization is that empty-handed glove gestures often get interpreted as target-oriented action gesture. Furthermore, when they shift to expressive movement forms, they are misread as signaling gestures.^14^

When comparing gloves to commonly available wearables, such as fitness armbands, myography bands, or watches with physiological sensors, the main difference is their contextual usage. Gloves for gestural music performance as shown in this article always form a compound aggregate with a software part, and are deployed in specific concert situations with the expressed intention of a musical performance. Wearable devices are mostly connected to other types of services, be it information transmission or physiological measurements. In conventional applications, wearables on the one hand may sense dynamics, hand muscles, spatial orientations, and physiological data such as blood pressure—these data are interpreted as activity such as steps with a quantitative metric or as physiological state, its analysis over long time spans providing the salient information. On-body sensing for media interactions, however, produces streams of movement-related data that are analyzed and applied to sound and media in real time in a direct adaptive action-perception loop (see Figure 2). Whereas gloves function as enablers of active connections, in an interaction paradigm, conventional wearables are worn as quiet observers or information conduits, in a capture-and-analysis paradigm. Both are using similar sensing mechanisms and affordances, but their goals, constraints, and applications differ significantly.

## Capture, Control, or Express?

Let us attempt to identify some underlying critical issues of wearable sensing and gesture-based interaction with computational processes. The interface between individual and world, movements, and gestures are defined by their intention, by their form, by their application, and by their wider context. Making things more difficult is the fact that not all of the key elements of movement can be readily accessed, and those that are accessible require a variety of approaches with different problem domains.

What is it we are trying to discover, describe, and understand? Is movement the core element we need to be investigating? Does computing necessarily mean single domain, univocal formalisms? Is formalization a necessary step in movement analysis?

If we consider that music making is one of the fundamental achievements of culture, then the technological practices possible today are an expression of the state of our world. As an exemplar and crystallization of our culture, some of the underlying issues and questions of our relationship to technology, art making, and the intersubjective exchanges are highlighted by these hybrid practices. As performers, we step into the liminal space (Turner, 1969, 1982; Mills, 2019) to create social situations of exploration, and for that purpose, we throw into the ring the full set of tools and techniques at our disposition. The following sections try to trace a path through a number of loosely connected topics that all have the potential to contribute to better understanding of what a contemporary music performance with sensors and interaction technology might be. Each tries to address one area of concern to, for example, glove-based electronic sound performance. Rather, the giving answers to these connective links and systematizing the problem space of movement, music, and performance research, a collection of questions if offered that may serve to map out domains that are still open for investigation.

### Fragmentary Replacements

We use technology in order to understand or alter our relationship with the body, movement, intention, and therefore meaning. However, this can only work in a state of suspended disbelief. In order to properly function with deeply entwined technological processes, such as performing with gloves, we need to ignore the fact that the tool is limited in its effect and that, by extension, it limits our capabilities of moving, seeing, hearing, and feeling. So, what, then, does the addition of computation bring to human movement, action, and gestural performance, their study, analysis, and application, for example, in a musical context? How does skin-tight overlaying of gesture with wearable sensing through gloves alter the performance?

Some of the fundamental functions of computers and their media output consist in the abilities to mirror, store–replay, and interconnect layers of information or data. In our case, however, it should not be forgotten that the data only partially represent the measured phenomenon, that is, the hand gesture, and that this representation in discrete units has an agency and impact of its own, which is not identical with that of the moving body (Stiegler, 2006).

In the context of living movement of bodies, the storage–replay functionality is interesting because it offers the opportunity to experience our body in close temporal proximity with the movement, gesture, or action from an outside, mediated perspective (Schnell, 2013). The illusion of a “real-time” response affords the engagement in an adaptive loop, where the organic and constructed structures enter into an relationship of mutual influence, that is, a mediated action-perception loop. This extension of the relationship between movement and data, between memory inscribed in the body and that stored in an apparatus, alters the perspective and understanding of movement and computing. However, the difference between human and machine memory should not be underestimated in this configuration: Whereas human *memories* are inscribed in the body and mind and remain tied to experience and are ever-changing (Bergson, 1889, p. 166, 1911), the status of *data* as stored memory changes according to its usage context (Simondon, 1958), but does not shift based on the experiential context, as human *memories* do.

Applying mathematical models to organic movements resembles a process of dissection and can lead to deeper understanding of human capabilities, for example, in biomechanical study of human (and nonhuman) motion and movement.^15^ This is the direction taken by methods that quantify and attempt to understand mechanical movements, for example, least-effort curves, economy of movement, and ergonomics (Eklund, 1997), as measured qualities that are perceivable or even beneficiary both for the mover and the viewer. This depends on the ability to read, measure, and capture human movement, which to some extent is always necessary when the task is to create, train, and optimize expertise. In this regard, simulation is one of the driving forces for the development of movement capture technologies, such as is used, for example, in cinema and computer games.

What about nonhuman movement? A different field of study synthesizes movements to make them look human, to provide a counterpart in mixed, yet artificial encounters. The field of robotics extends this to include nonhumanoid shapes and processes (Burdick, 1989; Knight and Simmons, 2015; Coupeté et al., 2016; Flash et al., 2019).

In the artistic context, the issue of computing with movement seems to be rather about capturing the ephemeral, unarticulated, and nonidentifiable elements of movement, in order to augment or extend the body’s capabilities, the artistic language, or the social, ritual, and scenographic situation. The store–replay capability, together with the capability of layering information, provides the advantage of applying computation to the arts: It complements, extends, and alters the mainly oral transmission of corporeal practice in performing arts (dance, music, and theatre).^16^ The capabilities for modeling and formalizing elements in algorithmic systems provide a tool for the creation of movements, choreographies, and instrumental gestures. Thus, the technological apparatus becomes a tool for augmenting existing movement, which poses the question: in order to do what?

One possible poetic answer would be: in order to provide unseen or unheard experience by “thickening” them, that is, by adding layers of additional materials, significations, and expressions, and thus shifting the artistic practice into a territory that is less well charted.

### Discrete Units

The problem with scientific discourse is that it slices up time and movement into isolated positions, the way a slide projector does (Deleuze, 1986). Science eliminates qualitative features of experience. It ignores *duration*, the qualitative element of time, and *mobility*, the qualitative element of movement (Guerlac, 2006, p. 68, my emphasis).

The most intriguing and delicate term used in the discourse about movement, its capture, and computation is the notion of *quality.* Apart from being the counterpart term to *quantity*, it is difficult to isolate and describe “movement quality” properly. Depending on movement practice and research focus, the dimension of quality varies wildly. Even so-called qualitative research, that is, psychological or social science methods that extract experience information and then treat it with mathematical or linguistic models, cannot provide a clear definition and therefore a computable basis for qualities of movement, gesture, action, or any other corporeal expression.

Wearable computing in general and glove-based gesture sensing in particular is no exception to this dilemma. The sensor values are discretized in time series, and the applied analysis models integrate the data streams as if they were neutral, yet valid representations of expressive movements.

Since Laban (2011) provided a systematization of living movements into effort,^17^ we know that qualities of movement may be perceived as their “inner aspects” and at least let us differentiate and identify movements with some semblance of intention and expressiveness. The categorizations implemented in his systematic analysis are aimed at being universal and are sufficiently general in order to be applied to many if not all movement descriptions, or so current applications suggest. When examining their scope and object, however, it is evident that these categories are mainly applied to free movement in space with the entire body, such as dance movements or whole-body gestures. Furthermore, the categories mix source and destinations effects, that is, the perception of intention (Proust, 2003), its manifested form within perception (Gallagher, 2005), and its affective and experiential impact (Tomkins, 1962).

Considering the least-effort paradigm for any optimized movement patterns (Viviani and Flash, 1995), for example, the performance quality of a given, choreographed movement sequence as it is performed by high expertise performers, we can but wonder as to how much *implicit bias* the notion of movement quality contains in this standardized system.

Between the sampled and quantized, frame-by-frame representation of a fluid movement and the perception of a fluid quality, both for the person executing the gesture and the person perceiving it in the other, there is a difference *in kind* of perception that cannot readily be bridged, if at all.

Bergson speaks of indivisible qualities that are the expression of intention. Experience, in his view, is based on representative as well as affective sensation, the former being measurable, whereas the latter is pure quality without reference to any external cause (Guerlac, 2006, p. 58); therefore, “intensity is situated at the point where the two currents meet” (Bergson, 1889, p. 54). Somatic practices and Somesthetics (Shusterman, 2008) emphasize awareness of the body during movement; the quality is evaluated according to the felt, not necessarily externally perceivable or measurable movement aspects. This poses the question of expertise, which is a large discussion in its own right (Schacher and Neff, 2016b).

### Hybrid Approaches

How to investigate performative gestures through wearable computing approaches to movement analysis? What kinds of answers can we hope to achieve with the methods that formalize movement into quantifiable number streams?

“Mixed Method Research” represents an attempt at bridging between quantitative and qualitative research methodologies (Johnson et al., 2007). It connects the measurement-based formalization and the descriptive explication of subjective experiences, both of which are “positivist” approaches based on the paradigm assuming that things are measurable and can be put into a formal mathematical, unequivocal representation or system. For example, in much of Music Psychology, the psychophysics approach is dominant: An experiment is devised to isolate the relationships between a stimulus and a reaction through the observation of behavior. This is intended to construct a piecewise map of how the human psyche and cognitive apparatus function.

The tension between inductive and deductive approaches and the scope of research influences the positioning and production of the outcome across the range spanning formalized-technical (Xenakis, 1992) and descriptive-hermeneutical (Gadamer, 1975) approaches. The context of research furthermore biases or predefines the type of questions generated from the outset and the kinds of outcomes produced at the closing of a research arc. This is as much a question of institutional demands, structure, and imposed quality criteria for research, as one of position and status of the individual researchers within the system.

Is there another way of doing research about the topics of embodiment and its perception in performing arts? Can research within a multiply defined field, which merges scientific, scholarly, and artistic approaches be fruitfully concentrated to address the big issues around body, movement, and cognition? Is an *experimental* as well as *experiential* approach possible, which spans from the direct, prelinguistic, and noncoded experience and its making (in arts), all the way to the analytical methods, which leverage advanced computation techniques and capturing technologies in real time? Can this approach produce a meaningful and unique perspective on movement research?

This configuration of topics contains disparate and contradictory levels of complexity in the domains of biology, cognition, psychology, physics, and so forth, but also culture, discipline (artistic), practice contexts, and social significations. The use of technology can, but does not have to be a factor in bringing to the foreground aspects along one of these lines. However, perhaps technology is merely an element of practice to be chosen and then developed in order to clarify, isolate, juxtapose, combine, and connect elements of the configuration that would otherwise be less directly connected. Understanding minute movements in relation to music performance, for example, through the capture, measurement, and subsequent visualization of kinematic data only became possible once motion capture technology became accurate and accessible enough to carry out this research (Jensenius, 2017). Capturing hand gestures through sensing gloves opens up a view on movement dynamics that otherwise would remain obscured by other perceptual interpretations.

### Research About Movement: How?

To research means to ask questions within a specific frame, while continuously altering the frame and continuously looking to repose the questions differently in light of the evolving perspective and insights. This fluidity and indeterminacy poses the methodological problem and raises the fundamental question of how to constrain the field of operations. The question is whether this is a necessary step in order to reduce the research area to a manageable size. Is not rather *uncertainty* and the difficulties inherent to delimiting fields part of the honest attitude of (slow) research (Stengers, 2018)? If reduction is pragmatically necessary, it should not eliminate the *cross-contaminating* and *fertilizing* aspects that only arise from a hybrid position: It should not be made simpler than necessary, to paraphrase a famous physicist.

A properly transdisciplinary position of research about movement and its systematization with or without technology is in need of a clear anchor to be able to extend beyond existing disciplines. What are such anchors and how do we integrate them into our methods and research plans? What institutional frames and conditions do we need in order to obtain the necessary space to work in this way? The principal challenge is how to circumvent the ever-increasing pressure to conform to standardized methods and schemata (Feyerabend, 1970) and carry out research in a slow enough manner to reach deeper insights than what is afforded by short project and study cycles (Stengers, 2011).

### Smooth or Striated

We come upon the two fundamental illusions of reflexive consciousness. The first consists in considering intensity as if it were a mathematical property of psychological states and not … the special quality, the nuance specific to these diverse states. The second consists in replacing concrete reality, the dynamic process that consciousness perceives, by the material symbol of this process arrived at its term. [This would be] to wrongly suppose that the symbolic image by means of which one represented the performed operation has been drawn by this operation itself in the course of its progress, as if registered by some recording device (Bergson, 1889, translated by Guerlac, 2006, footnote, p. 87).

Human thinking, that is, conscious, discursive, propositional thinking, is based on units of meaning, words, and sentences, and the concepts and notions attached, mobilized, enabled through and to them. The structure of language, and scientific language in particular, presupposes a static connection of signification to objects, ideas, concepts, and experiences, in order to be able to operate in a repeated, stable, and thus sense-accumulating manner. Since the relationship between the elements in this system is standardized, it has the power to become carrier of information and enable communication across the interindividual divide and across the time spans and spaces of and in between cultures (Vygotsky, 1962). All of these aspects apply to mathematics in an even more strict sense; computation as a concrete implementation of mathematical principles is an expression of this need to subdivide and standardize.

When we consider the analogical nature of being human—our body and our experience are in continuous exchange with the environment, with other humans, in social and cultural domains—a different type of organization becomes evident. In it, layer upon layer of organic adaptation, energy signatures, phrases, or what Luria (1973) (cited in Sheets-Johnstone, 2003) calls the kinetic melody, and codependent processes of excitation and inhibition occur. These do not appear as separate and clearly delimited domains or processes, and this is where we encounter our fundamental dilemma: They are intertwined, mixed-up, codependent, and in continuous flux, changing their relations between themselves and to the outside, the environment. Cognition arises from the adaptive loop in which the organism evolves, grows, and learns (Varela et al., 1974; Maturana and Varela, 1980). Our perception is focused on the environmental link: The affordances (Gibson, 1977) of the situation are read, the intent of the other is interpreted (Decety and Chaminade, 2003), and the cultural codes are deciphered (Geertz, 1973).

Wearable and transparent sensing with glove interfaces may become probes into the otherwise opaque interrelation between body and environment. Moreover, in the cultural situation of music making, they may not only enable but also uncover intrinsic connections between intention, gesture, and sonic outcome. With their influence on the link and adaptive loop between body and instrument, they may interfere in but also catalyze this complex mesh of influences.

### Ripples in the Manifold

From the cognitive perspective, our movements and gestures seem like mere artefacts of our engagement with a much thicker, richer, and wider frame of reference. Perhaps they are just ripples on the surface of our actions within and toward the world. They carry existential, factual, whimsical, or even poetical signs and signals from our intention out into our environment. Effort, expressivity, or quality then become codes for a specific manner of execution, which is both effective in terms of ergonomic flow and efficient as carriers of affect and meaning (Gabrielsson and Juslin, 2003).

The dilemma and paradox of movement computation lies therefore in the necessity to think in terms of discretized units of meaning (and measurements) with regard to a fundamentally heterogeneous and indivisible aspect of human nature and culture. The sampling theorem and the standard procedure of science to discretize its object of study fail to account for the fact that human experience and its expressions cannot be subdivided into equal parts to be subjected to rules and algorithms. It is true that sampling enables the creation of outlines of, for example, movement trajectories and that time series of equidistant key frames enable to calculation of functions that can represent the measured phenomena in mathematical ways.

The question is how to articulate these aspects of the manifold complex (Held, 2003) represented by the body, much less how to carry out systematic work on this indivisible entity through the use of technology? Clearly, there is a gain to be obtained from applying computation to sampled, key-framed captures of movement, be it as work on media, transformations in the way we perceive movement, or as methods for bringing to the surface otherwise hidden aspects and parameters of movement. How much this represents anything approaching human experience and affective meaning is another matter altogether.

### Particle and Wave

Ever since Heisenberg formulated the uncertainty principle and Schrodinger put a cat in a box, we also know that phenomena in the physical world, albeit on an atomic level, do not exist and occur only in a *single* modality (Popper, 1935, pp. 243–247). Quantum *entwinement* furthermore tells us that the classic laws of cause and effect, of energy conservation, and of reversibility of physical phenomena are not universal, and that there are domains where we have to deal with a complex duality of states, an ambiguity that cannot be resolved with deterministic logic and argument (Barad, 2007).

Human behavior and as a subset of this, movements and gestures, in particular in performing arts, should perhaps be considered to exhibit the wave–particle duality in a *translated manner.*

Perhaps we can use the *particle* as model for the discretized movement that gets captured in data points in the time series generated by a wearable device such as a sensor glove. This modality may be useful for describing and formalizing, through the slices and still frames needed to operate on clearly delimited and standardized units of measure.

*At the same time*, we should consider human perception and its ability to apprehend a heterogeneous mass of stimuli present in human movement, gesture, as well as culturally bound behavior, without the need to “chunk” it (Godøy et al., 2010). This continuous flow of information then perhaps behaves more like the *wave* phenomenon, as an energy traversing a medium.

Can these two fundamentally different ways of apprehending human and living movement be reconciled? How must a method for researching this domain be structured to do justice to the dual nature of inner and outer impact and significance? How to bridge homogenizing formalism and the heterogeneity of experience?

### Productive Dilemma

The paradox of movement analysis with computing tools should be considered a productive dilemma. If we accept the fact that human movement comprises action, gesture, and intention, as well as perception and resonance, that it stretches across numerous facets of our experience, and that *research as performance* with technology on this topic means implicating multiple disciplines and creating new methodologies, then perhaps the most constructive approach is to create a larger frame for research, which productively combines, but also critically problematizes, the different approaches that it includes.

How can the scientific method necessary for working with technology and mathematical formalism, the scholarly reflection leading to theory and critical thinking, the mixed-method psychological experimentation aimed at understanding hidden aspects of human perception and behavior, and the experience-based investigation come together and produce a meaningful outcome? How can hidden, inner processes (Kozel, 2015), perceptions, and affects be combined with the outer manifestations of human intentions through movement, action, gesture, and performance? Why do artistic performance practices provide such a rich and singular field in which to study precisely the connection between inner and outer aspects of “living” human movement? Can a methodological inquiry that originates from reflection about deciphering the cultural, cognitive, and psychological implications of an artistic practice be brought to a sufficiently differentiated level that a contribution to epistemological thinking can be attained? These and many other questions arise from this research perspective. The case of gestural music performance with gloves as wearable links to computational processes may serve as a lens through which to investigate this thick bundle of issues.

The theory of blended spaces may provide a guide here as well, as it postulates that: “The essence of the operation is to construct a partial match between two input mental spaces, to project selectively from those inputs into a novel “blended” mental space, which then dynamically develops emergent structure” (Fauconnier and Turner, 2003).

Unifying research about the inside perspective and the outside manifestation of bodily expressions, be it as movement, action, and gesture, or as intention, effort, and affective impact, should thus open a fruitful avenue for research. A nondualistic, integrative approach would be aimed at uncovering both the primary experience of movement through its performance (Kozel, 2015) and processes (Seibt, 2018), as well as the epistemic gains that the perspective could afford. By operating at the intersection of these perspectives and by using methods that span across disciplines, the challenge is to create a rich enough “object” of research to do justice to its “thickness” (Geertz, 1973). If the “remediations of [a] fleeting live movement [in order] to make it a reproducible artefact” (Noman, 2014) can tie the inner to the outer domain, what could a *cross-mediation* in repeated processes across methods and practices produce?

A circular, iterative approach across perspectives, ranging from the data-driven quantitative methods, the experience-based qualitative analysis, to investigations of experience, has the potential to create an overarching, intersecting object, more appropriate to the study of the complexity of human expressive movement.

## Augmented Spaces

Wearable interaction using on-body, hand-sensing gloves for musical performance produces a split, multilayered attention both for the performers and the audience. Only with extended training and a long-term stable configuration can embodied preconscious behavior patterns, that is, well trained and ingrained body schemata, be attained—without having to adapt them to accommodate the technically defined affordances and action spaces of the sensing devices. With the aid of a tool or instrument relationship that builds on metaphors of interaction this integration may be achieved. However, the measurements and data captures obtained from high-dimensional hand physiognomy lead to complex control spaces, which need to be reduced and remapped to habituated actions and processes with natural world cultural givens (i.e., related to habitual tool use). The challenge is to develop mappings that allow reconstructing an action space that feels natural. Achieving this across the superposed merged surface of sensing gloves is not trivial. It presupposes the ability to surpass real-world interaction metaphors as well as purely functional instrumental control actions.

Wearable interfaces tie the body to comparatively narrow affordance spaces: Contrary to control interfaces, the wearable relationship superimposes technical structures onto the bodily space, which, once put into place, cannot easily be dissociated, only modulated.

Gloves for on-body sensing in musical interactions generate an augmented gesture space that produces a semantic or functional enrichment of an already densely packed domain of action, interaction, expression, and signification. The close proximity of the glove interface with the hands represents a merging of body space and instrumental (control) space. Thus, the immediacy of wearable sensing without intermediary external objects produces a kind of fusion between action space and affordance space of the hands and that of the instrument.

The cognitive, social, and cultural facets of performing are added to this fused space, with the result that the primary impact of these performances occurs on an affective, prereflective level, where the aforementioned domains come together as an organic whole. When therefore considering the number of perspectives and dimensions that are at play in these performance situations, it becomes evident that successful performances are only achieved by artists with much training and a capacity to imprint and make subconscious the complex operations of their apparatus. In this configuration, the focus of the performer does not need be directed at a visual display or control surface of any kind, freeing the gaze and therefore creating a more immediate relationality with the audience. The instrumental interface is superimposed to the hands and arms and conforms through the sensing modalities and the mapping to their specific affordances. Through this closeness, the instrumental performance gets encapsulated or made invisible and transparent.

Compared to conventional media or music performance, the artists sacrifice the legibility and clarity of a traditional musical instrument. Yet, through the skin-tight connection with part corporeal and part invisible digital instruments, they gain the freedom to engage with empty hands and to openly face the audience in an unmediated and shared space of affective encounter.

## Data Availability

All data and media materials that are available are mentioned within the article’s main text with links to their online repositories in footnotes.

## Footnotes

1 Available at https://www.grame.fr/productions/light-music (all URL’s last accessed 1 February 2022).

2 Available at https://mimugloves.com/.

3 Here is a video of his TED talk. Available at https://www.ted.com/speakers/onyx_ashanti.

4
http://www.steim.org.

5 A program note for his concerts said: “Image: Balinese dancers, old European magicians, boxers and conductors connected by wire … Sound: If music … was about cyclical activities of rowers … then ‘Hands’ is about rowing itself” (Vasulka, 1986, p. 10).

6 DMI: a term coined and applied in the NIME community.

7 A web page and videos can be found online: available at http://steim.org/2009/10/remembering-mw/ (1993); https://vimeo.com/1203648 (1994); https://vimeo.com/1203709 (2004); and https://vimeo.com/1203648.

8 A documentary film about Laetitia Sonami was made by filmmaker Rsitoy in 2015 and is accessible here: Available at https://www.rsitoy.com/laetitia-sonami-film.html; https://vimeo.com/ondemand/earfilm.

9 Was https://www.thalmic.com/en/myo/ now defunct and acquired by Google. Available at https://www.bynorth.com/; demo video: https://www.youtube.com/watch?v=A8lGstYAY14.

10 Suspensions, for piano and EMG interface, is a work by Atau Tanaka for solo pianist and electronics. Available at http://helicotrema.blauerhase.com/atau-tanaka-2/.

11 Myogram 2015—Performance by Atau Tanaka. Available at https://mindthefilm.co.uk/projects/myogram-performance-by-atau-tanaka/.

12 Available at https://www.jasch.ch/lha_rgyal_lo.html; https://www.jasch.ch/codespace.html; and https://www.jasch.ch/opticalidentity.html.

13 For a collection of videos documenting the evolution of this piece, please see https://www.researchcatalogue.net/view/269265/272400 and https://www.jasch.ch/island.html.

14 Or as references to dance traditions with codified hand movements, such as Indian Kathak and Kathakali, Spanish Flamenco, or Balinese temple dances.

15 Can this perhaps be differentiated between the physical and unintentional versus the intentional and expressive?

16 In particular, in relation to choreographic transmission, motion captured performances can also serve as archival format (Fernandes and Jürgens, 2010; Shaw, 2011; deLahunta and Jenett, 2017).

17 “In order to discern the mechanics of motion within living movement … it is useful to give a name to the inner function originating such movement. The word used here for this purpose is *effort.* Every human movement is indissolubly linked with an effort, which is, indeed, its origin and inner aspect” (Laban, 2011, p. 20).
